# Supplementary material for: A scabies outbreak in the North East Region of Ghana: The necessity for prompt intervention
Source: PLoS Negl Trop Dis. 2020 Dec 22;14(12):e0008902. doi: 10.1371/journal.pntd.0008902 (PMC7787682; doi:10.1371/journal.pntd.0008902)
Supplement: S1 Text — (DOCX) [file pntd.0008902.s001.docx]

**SCABIES OUTBREAK QUESTIONNAIRE**

BASIC DEMOGRAPHY FORM

1. Study ID
2. Date of Visit
3. a. First Name b. Last Name
4. Is this questionnaire school based or community based?
5. School based b. community based
6. Are you the first one to be interviewed in this household?
7. Yes b. No
8. If not the first to be interviewed, what is the study number of the first person to be interviewed in your household?
9. What is your address? (if school going, address of family)
10. Relative address (identifiable landmark if possible)
11. What is your District?
12. What is your Region?
13. What is your Phone number?
14. What is your date of birth?
15. What is your age?
16. What is your sex?
17. Female b. male
18. What is the number of people in your household?
19. How many people in the household have complaints of an itch?
20. How many persons in the household are participating in the questionnaire today?
21. Do you sleep in a bed alone?
22. Are you going to school?
23. What is your level/class at school?
24. What is the name of your school?
25. How long does it take you to walk to school? In minutes?
26. Which work do you do?
27. Farmer b. trader c. hairdresser d. mechanic e. teacher f. chief of village g. other
28. If other, which work?
29. Comments

**HISTORY (SKIN)**

1. Do you currently have an itch?
2. How long have you had this itch? (in days)
3. Do you currently have a rash?
4. How long have you had this rash? (in days)
5. Did you have contact with the following in the past weeks?
6. House contact with itch
7. School contact with itch
8. House contact with scabies rash
9. School contact with scabies rush
10. Comments

**SKIN EXAMINATION**

1. Skin examination; burrows on the participants’ skin?
2. Yes b. No
3. Skin examination; rash typical for scabies on exposed areas?
4. Yes b. No
5. Is it crusted rabies?
6. Yes b. No
7. If skin lesions visible is suspected scabies, where were they located?
8. Fingers
9. Finger webs
10. Wrists
11. Hands
12. Forearm
13. Toe webs
14. Feet
15. Leg
16. Face
17. Neck
18. Scalp
19. Other
20. If other, where?
21. Comments

**OTHER SKIN DISEASES AND COMPLICATIONS**

1. Does the participant have any skin disease or abscess?
2. Yes b. No
3. Does the participant have an ulcer or wound?
4. Yes b. No
5. Does the participant have signs of impetigo?
6. Yes b. No
7. How severe is the impetigo?
8. Very mild, 1 to 5 lesions
9. Mild, 6 to 10 lesions
10. Moderate, 11 to 49 lesions
11. Severe, 50 or more lesions
12. How was the impetigo treated?
13. With antibiotics
14. Not treated yet
15. Other
16. Which antibiotic was used?
17. If other, how?
18. Does the participant have signs or have a history of eczema?
19. Yes b. No
20. Does the participant have post trauma depigmentation?
21. Yes b. No
22. Does the participant have signs of vitiligo?
23. Yes b. No
24. Does the participant have signs of a fungal skin infection?
25. Yes b. No
26. Does the participant have an abscess?
27. Yes b. No
28. How was the abscess treated?
29. With antibiotics
30. With surgery/ small incision
31. Not treated yet
32. Other
33. Which antibiotic was used?
34. The participant with active disease is referred for further treatment?
35. Yes b. No
36. The participant collected urine?
37. Yes b. No
38. Comments

**TREATMENT**

1. Have you received treatment for scabies in the past two months?
2. Yes b. No
3. Why were you treated?
4. To treat own skin problems
5. No skin problems but close contact with scabies
6. Did you have any of the following when you were treated?
7. Itch
8. Rash
9. House contact with itch
10. House contact with scabies rash
11. School contact with scabies rash
12. No
13. What drugs did you receive?
14. Benzyl benzoate
15. Permethrin
16. Ivermectin
17. Other
18. If other, what treatment was it?
19. Did you use herbal preparation?
20. Yes b. No
21. How and when did you use the other drug or herbs?
22. How frequently were you treated with benzyl benzoate? (number)
23. When did you use the drug for the first time? (date)
24. When did you use the drug for the second time? (date)
25. When did you use the drug for the third time? (date)
26. How frequently were you treated with Ivermectin? (number)
27. When did you use the drug for the first time? (date)
28. When did you use the drug for the second time? (date)
29. When did you use the drug for the third time? (date)
30. Was any household member treated for scabies at the same time as you?
31. Yes b. No
32. How many household members (apart from you) were treated at the same time? (number)
33. How many of them are going to this school?
34. How many of these household members had complaints of scabies when they received treatment? (number)
35. On the day you were treated, did your family do the following activities?
36. Wash clothes used in three days before treatment
37. Wash all bedsheets and bed linen
38. After applying drug, dress with clean clothes
39. Other cleaning or hygiene precautions
40. None
41. Which other cleaning activities or hygiene precautions?
42. Did you have something to eat in the two hours before or after taking the drug?
43. Yes b. No
44. Did you bath before applying the ointment?
45. Yes b. No
46. Did you cut your nails before using the ointment?
47. Yes b. No
48. On which body parts did you apply the ointment?
49. Only body parts with rash or itch
50. Full body apart from head
51. Other
52. If other, how?
53. For how many hours did you leave the ointment on your skin? (number)
54. When using the ointment, how long did you use it?
55. Applied once, ointment on body for one day
56. Applied it once, ointment on body for two days
57. Applied it twice, ointment on body for two days, 1 application per day
58. Other
59. If other, how?
60. Comments
