# Supplementary material for: A scabies outbreak in the North East Region of Ghana: The necessity for prompt intervention
Source: PLoS Negl Trop Dis. 2020 Dec 22;14(12):e0008902. doi: 10.1371/journal.pntd.0008902 (PMC7787682; doi:10.1371/journal.pntd.0008902)
Supplement: Attachment — Submitted filename: Response to Scabies review comments.docx [file pntd.0008902.s002.docx]

25^th^ June, 2020

The Editor-in-Chief

PLOS Neglected Tropical Diseases

Dear Sir,

**Re: Decision on your PLOS Neglected Tropical Diseases submission (PNTD-D-20-00305) - [EMID:a2747443bf3c1ce6] "A scabies outbreak in the North East Region of Ghana: the necessity for prompt intervention"**
 
My co-authors and I will like the editors and reviewers for the feedback on our manuscript. We have taken note of the review comments and wish to respond as follows:

Reviewer's Responses to Questions

Key Review Criteria Required for Acceptance
As you describe the new analyses required for acceptance, please consider the following:
Methods
-Are the objectives of the study clearly articulated with a clear testable hypothesis stated?
-Is the study design appropriate to address the stated objectives?
-Is the population clearly described and appropriate for the hypothesis being tested?
-Is the sample size sufficient to ensure adequate power to address the hypothesis being tested?
-Were correct statistical analysis used to support conclusions?
-Are there concerns about ethical or regulatory requirements being met?
Reviewer #1: Please see Summary and General Comments!
Reviewer #2: (No Response)
Reviewer #3: The population is partially described - if possible the authors should give an estimate of the number of communities and households in the region.
It is unclear as to how representative the sample of 283 patients is of the whole population (This should at least be acknowledged / or discussed in the discussion)

Response: We have included a section on study limitations which reads as follows:

‘This was an opportunistic assessment that was undertaken in the context of investigation of an outbreak situation in Northern Ghana. The sample size of this study is relatively small compared to the population of the entire district. Even though there may be differences in scabies prevalence between communities in the district, due to the close person-to-person interactions between communities (resulting from close extended family ties), it is unlikely that such a percentage would be lower than 10% (MDA indicated if higher) considering the high percentage of scabies found in the communities studied. The students studied come from different communities across the district. Furthermore, the district health team ended up providing MDA across the district and did not observe communities without scabies problems. This study provides useful data on scabies burden and could form the basis for guiding future research in Ghana and West Africa where there is a dearth of data on scabies prevalence.’(Line 320-333)

We had also acknowledged in the methods section that ‘the team started their activities in the communities and a boarding school where local authorities reported the burden to be high’ (line 96-98).

Results
-Does the analysis presented match the analysis plan?
-Are the results clearly and completely presented?
-Are the figures (Tables, Images) of sufficient quality for clarity?
Reviewer #1: Please see Summary and General Comments!
Reviewer #2: (No Response)
Reviewer #3: data analysis mostly matches plan - some comparative statistics provided - no odds ratios provided with p-values, not clear in methods how these comparisons were made

Response: We have included a section on ‘statistical analysis’ to address the concern raised by the reviewer (line 141-148).

Conclusions
-Are the conclusions supported by the data presented?
-Are the limitations of analysis clearly described?
-Do the authors discuss how these data can be helpful to advance our understanding of the topic under study?
-Is public health relevance addressed?
Reviewer #1: Please see Summary and General Comments!
The limitations of analysis are not clearly described.

Response: We have added a section on study limitations as indicated in response to reviewer 3 above (line 320-333).

Reviewer #2: (No Response)
Reviewer #3: The conclusions are not fully supported by the data - particularly regarding the need for MDA (which was not feasible in this case) and the reason for ongoing transmission being related to the lack of treatment of contacts.
Limitations and strengths are not discussed
The implications of the findings are not fully explored.

Response: We have added a section on study limitations (line 320-333). We have provided additional information in the discussion on MDA – line 300-319 (see response to comment 16 by reviewer 2 below).
As per the recommendation of this reviewer, we revised the sentence (in the abstract and conclusion) to now read: ‘Mass drug administration maybe an effective strategy in such settings to contain outbreaks.’ (Line42-43)

Editorial and Data Presentation Modifications?
Use  this section for editorial suggestions as well as relatively minor modifications of existing data that would enhance clarity. If the only modifications needed are minor and/or editorial, you may wish to recommend “Minor Revision” or “Accept”.

Reviewer #1: Please see Summary and General Comments!
The topic of the article is highly relevant, but the presentation has too many weaknesses and needs to be rewritten.

Response: We thank the reviewer for the useful comments and suggestions. We have made the revisions as recommended by the reviewers.

Reviewer #2: (No Response)
Reviewer #3: The authors summarise the importance of scabies and the data available to date from Ghana. The use of the IACS criteria is relatively novel and demonstrates the utility of this tool in the field setting. The authors demonstrate a high prevalence of scabies in the setting of an regional ourbreak, adding to limited national data on prevalence of scabies. There is discussion the potential role of MDA and limitations of this approach.

The methods and results should provide a clearer picture of how representative the sample of 283 participants is if possible. The discussion should be expanded to discuss the use of IACS criteria in the context of previous studies, the limitations of the IACS criteria, the strengths and limitations of the study and implications for future research.

Response: We have revised the manuscript to address the concerns as detailed in other responses to reviewers below (see response to reviewer 3 below regarding IACS criteria and response to reviewer 3 regarding study limitations above).

The discussion on MDA is of interest but perhaps a more broad discussion of other available strategies, including limitations (particularly as MDA was not feasible in this instance).

Response: We have revised the discussion to reflect the reviewer’s comment. We have added a section discussing the strategies for MDA with permethrin and ivermectin as detailed in manuscript (line 300-319) and in other responses to reviewers 2 and 3 below.

Abstract
- separate methodology and results

Response: This section is presented as per requirements of the journal. No changes done
- revise statement on mass drug administration - perhaps "maybe an effective strategy in such settings to contain outbreaks"

Response: Done as suggested (line 27-28)
- line 24 - change "remanifestations" to "recurrence and ongoing community spread"

Response: Done as suggested (line 25-26)
Author summary
- line 31 - change "huge" to "large"

Response: Done (line 33)
- line 33 - change "of" to "complicating scabies can lead to significant complicatiosn including..."

Response: Done (line 35-36)
- line 38 - change "remanifestations" as above

Response: Done (line 25-26)
- line 40 - revise statement on mass drug administration as above

Response: Done (line 42-43)
Introduction
- line 69 - reference?
Response: Done. We have provided references as requested (line 72).

Methods
- what is the estimated population of the area examined? (if available)

- how many communities were examined? what is the estimated size of the school?

Response: See response to reviewer 3 above.

- line 101 - dose this mean - all houses in each community were visited and ?all members of the household were invited to participate?

Response: We visited all houses within the communities and invited all persons present at the time of the visit to partake in the study. While this might be impossible in an urban setting, it is important to understand that the study area is in rural Ghana. Here, most of the communities are generally small sized settlements which farmers and their close relatives set up and reside in. There are (compared to urban areas) relatively few densely populated houses within such communities. Each family may have a house that usually consists of 2-3 huts/ living quarters/rooms which the entire household reside in.

- how was consent obtained for school children?
Response: In addition to the written permission from the local health authorities, the School authorities also gave permission for the conduct of the study. School children provided assent for their inclusion. Within the communities, parents/ guardians of children included in the study provided consent for their inclusion. We have revised the ethics section to include the statement (line 152-154) ‘In the school, in addition to obtaining permission from the school authorities which informed parents about the research activities, the school children who were < 18 years provided assent for their inclusion in the study’.

- how many team members conducted assessments and was any specific training required to perform IACS assessment?
Response: Five team members (YAA, JA, EA, KOA and MF) conducted the field assessments. Two team members (YAA- a Senior Specialist in Infectious Diseases and JA- an experienced Public Health Specialist) did the initial evaluation of the IACS tool for scabies diagnosis and subsequently provided training on the use of the tool to the other members of the assessment team. All trainings were under the supervision of ROP, T-vdW and YS who are Professors of Infectious Diseases with many years of experience in the field.

We have included in the methods the following statements: ‘The assessment team consisted of medical doctors with clinical experience diagnosing scabies based on earlier activities in infectious diseases and/or public health. In addition, a supplemental training program on the diagnosis of scabies, impetigo and other locally common skin conditions as well as the use of the IACS criteria for scabies diagnosis was provided to team members.’ (line 98-102)

- include in statistics how comparisons were made (between treated and non-treated patients?)
Response: We have clarified this in the section on statistical analysis which reads: ‘We conducted descriptive and inferential statistical analyses to present the data of the outbreak. Categorical variables were expressed as frequencies and proportions; and results for continuous variables were expressed as median and interquartile range (IQR). The severity of scabies and impetigo in the earlier treated and untreated groups were compared using the Mann-Whitney (MWU) test. The Relative Risk (RR) of impetigo in participants with or without scabies was calculated with 95% CI. Statistical analysis was performed using IBM SPSS statistics Version 20 (IBM Company, Armonk, NY, USA).’ (Line 141-148)

Results
- what proportion of the population does the 283 people represent? (appreciate there may not be readily available census data?)

Response: Please see response to reviewer 3 above.
- line 158 - provide odds ratio + 95% CI with P value 0.063

Response: The comparison has now been done using the Mann-Whitney U test and now reads: ‘The participants who were treated in the past months showed similar scabies severity compared to the untreated participants (MWU, p = 0.068).’ (Line 191-192)

- line 159 - provide odds ratio + 95% CI with P value 0.115

Response: The comparison has now been done using the Mann-Whitney U test and now reads: ‘Impetigo seemed less common among previously treated scabies participants; however, this was not statistically significant (MWU, p = 0.25).’ (Line 193-194)

- data on age stratified prevalence would be interesting if available, also gender differences
Response: Please see response to reviewer 2 below.

Discussion
- line 177 - 87% contradicts earlier statement on line 172 (0.2% to 71.4%)

Response: We thank the reviewer for drawing our attention to this. We have revised the section accordingly and it now reads ‘The scabies prevalence worldwide varies widely and depending on the population studied ranges from 0.2% to 87% (16, 21) with the highest prevalence in the island nations of the Pacific and countries in Latin America.’ (Line 208-210)

- line 208 - reference? Cochrane review on treatment comparison <https://onlinelibrary.wiley.com/doi/abs/10.1002/ebch.861>
- may be helpful to give previous figures (percentage response from clinical triasl)
- ?discuss difficulties with compliance

Response: We have inserted a reference here as recommended by the reviewer. We have also addressed the issues regarding compliance with benzyl benzoate. The section now reads: ‘One possible explanation could be that benzyl benzoate is less effective for the treatment of scabies compared to other treatment options like ivermectin or permethrin (6). Benzyl benzoate administered topically as a 25% solution may cause skin irritation especially in younger children and this may negatively impact compliance with therapy. In Ghana, the standard treatment guidelines requires application of benzyl benzoate over the whole body (except the face) twice and left overnight on two consecutive nights. The first application is done after a warm bath with the application repeated the next day (without a bath) and washed off 24 hours later. It is plausible that persons receiving the treatment did not fully adhere to the instructions on the use of benzyl benzoate.’ (Line 268-277)

- line 208 - yet "the main reason" should be revised -  the data does not completely support this conclusion - something like "the lack of treatment of household contacts is potentially a major factor leading to re-infection of cases and ongoing community transmission."

Response: The suggested revision has been done. The section reads: ‘Contacts of cases are usually advised to treat themselves at the same time as the case in order to reduce the risk of re-infection (5, 33-35). The lack of treatment of household contacts is potentially a major factor leading to re-infection of cases and ongoing community transmission.’ (Line 277-280)

- line 210 - discuss time to response following treatment - is it possible that some of these patients had 'resolving' scabies (e.g. those treated within 1-2 weeks of assessment)

Response: We have inserted the following sentences: ‘The interval from scabies treatment to response is variable. The rash and itch may persist for up to 2 weeks after treatment. Patients treated in the preceding 2 weeks might still have symptoms thus making it difficult to distinguish re-infections from primary treatment failure (5, 7, 34).’ (Line 285-288)

- discuss rationale for use of IACS criteria - health care worker recognition of scabies may be poor? <https://www.ncbi.nlm.nih.gov/pubmed/28671945> - laboratory diagnosis is impossible in most settings
Response: Done. We have addressed this by inserting the following: ‘Scabies and impetigo infections are reported to be under-recognised and hence under-treated by clinicians (1, 26) as laboratory diagnosis is impossible in most settings (1). The use of the IACS criteria for diagnosis may result in improvements in the recognition and treatment of scabies infections by clinicians; this will be important to reduce the high burden of scabies infection and subsequent sequelae among populations where scabies infection is endemic.’ (Line 244-248)

- discuss previous studies to report on IACS criteria utility - where does this study sit

Response: We have stated in our discussion section (line 241-251) that: ‘The 2018 IACS criteria of scabies consist of three diagnostic categories (confirmed, clinical and/or suspected scabies) (19). The clinical and suspected categories are practical for use, help to standardize reporting on scabies, and can easily be applied in field surveys like ours. Scabies and impetigo infections are reported to be under-recognised and hence under-treated by clinicians (1, 26) as laboratory diagnosis is impossible in most settings (1). The use of the IACS criteria for diagnosis may result in improvements in the recognition and treatment of scabies infections by clinicians; this will be important to reduce the high burden of scabies infection and subsequent sequelae among populations where scabies infection is endemic. The IACS criteria proved its utility when used for a school survey in the Solomon Islands (10). The current study has further demonstrated the practicality of using the IACS diagnostic criteria in a field survey even in a resource limited setting in sub-Saharan Africa.’

- discuss rationale for selecting representative population in schools (vs whole school)

Response: This was an opportunistic assessment that was undertaken in the context of investigation of an outbreak situation in Northern Ghana. Due to the constraints of time and limited resources, we could not undertake a full scale evaluation of the entire population. The sample size of this study is relatively small compared to the population of the entire district. Even though there may be differences in scabies prevalence between communities in the district, due to the close person-to-person interactions between communities (resulting from close extended family ties), it is unlikely that such a percentage would be lower than 10% (MDA indicated if higher) considering the high percentage of scabies found in the communities studied. The students studied come from different communities across the district. Furthermore, the district health team ended up providing MDA across the district and did not observe communities without scabies problems. We have emphasised this in the section on study limitations (line 320-333).

what if any treatment was provided to cases / contacts

Response: Cases and contacts who were identified during this assessment supplied with benzyl benzoate for treatment as recommended by the treatment protocol in Ghana. We have inserted in the methods section (line 138-139) the following statement: ‘Benzyl benzoate was supplied to participants with scabies and their contacts as per standard protocol in Ghana.’

- discuss limitations and strengths of the study

Response: We have added a section (line 320-333) which reads: ‘This was an opportunistic assessment that was undertaken in the context of investigation of an outbreak situation in Northern Ghana. The sample size of this study is relatively small compared to the population of the entire district. Even though there may be differences in scabies prevalence between communities in the district, due to the close person-to-person interactions between communities (resulting from close extended family ties), it is unlikely that such a percentage would be lower than 10% (MDA indicated if higher) considering the high percentage of scabies found in the communities studied. The students studied come from different communities across the district. Furthermore, the district health team ended up providing MDA across the district and did not observe communities without scabies problems. This study provides useful data on scabies burden and could form the basis for guiding future research in Ghana and West Africa where there is a dearth of data on scabies prevalence. The IACS criteria for standardisation of scabies diagnosis was easily and practically applied in our field survey in a resource limited setting in rural Ghana.’

Summary and General Comments
Use  this section to provide overall comments, discuss strengths/weaknesses of the study, novelty, significance, general execution and scholarship. You may also include additional comments for the author, including concerns about dual publication, research ethics, or publication ethics. If requesting major revision, please articulate the new experiments that are needed.

Reviewer #1: Scabies is one of the world’s commonest afflictions and despite that a neglected disease. As such it warrants public health interventions and a global control programme. There are still, however, several key operational research questions to be addressed, e.g. prevalence data, before mass drug interventions could be outlined. The present article dealing with a local scabies outbreak in Ghana should therefore be of great scientific interest and merit publication in PLOS. Of the 11 authors, seven are from Ghana and three from the Netherlands. The affiliation of Michael Frimpong is lacking.

Unfortunately, I cannot recommend acceptance of the article as it now stands. Generally, it needs more editing, more consideration for details, better structure and focus, less repetitions and better information for the readers to be able judge on the internal and external validity.
The incitement for the data collection and the study was a reported outbreak in northern Ghana communities. Despite individual treatments the transmission of scabies had not been appreciably reduced. It would be interesting to know whether Ghana has standardised instructions for individual case-management of scabies and impetigo.

Response: We have inserted the affiliation of Michael Frimpong.

We have revised the manuscript in accordance with the suggestion and comments from our esteemed reviewers. We have also included in the discussion, the standardised instructions on the use of benzyl benzoate. The section (line 272-275) reads: ‘In Ghana, the standard treatment guidelines requires application of benzyl benzoate over the whole body (except the face) after a warm bath with the application repeated the next day (without a bath) and washed off 24 hours later.’ We have also inserted the sentence (line 140) on impetigo management as follows: ‘Impetigo was treated as per standard protocol in Ghana’.

Some specific comments
The Introduction should give the readers information on scabies generally and then zoom in on the general situation in Ghana. We need to understand a bit about how the health care operates and what is meant with a district, a municipality, a community, a village. A figure could be of help.
The introduction should end with clear stated aims. “In this study we report ............” is all too vague. Using a descriptive epidemiological Methodology the authors report prevalence data and assess the impact of earlier individual treatment on their findings by applying the IACS diagnostic criteriae.

Response: The aims of the study have been clearly stated in the manuscript (line 84-86) as follows: ‘In this study we report on findings from that outbreak, the effect of earlier individual treatment strategies on clinical findings and the use of the International Alliance for the Control of Scabies (IACS) diagnostic criteria for scabies for a field survey on scabies in rural Ghana.’

We have replaced municipality with district throughout the manuscript to ensure uniformity.

In the Subjects and Methods section we need to know more about the Site: which community/communities are involved and their populations, or if census data are not available, at least an estimate thereof. Number of health clinics? Schools, boarding schools? The sentence beginning on line 90: “The district shares boundaries ..........” should be left out.

Response: Please see response to reviewer 3 above.

In addition, the sentence ‘The district shares boundaries with Talensi and Nabdam Districts, Bawku West and Garu-Tempane Districts to the north, Bunkpurugu/Yunyoo to the east, Gushiegu and Karaga Districts to the South and West Mamprusi to the west’ has been deleted as suggested by the reviewer.
Line 94: are “structures” the same as houses?

Response: We have replaced ‘structures’ with ‘rooms’ (line 93)

The data collection team need to be better presented in a paragraph of its own. Criteria for their recruitment? How many were they? Were they especially trained for the task?

Response: Five team members (YAA, JA, EA, KOA and MF) conducted the field assessments. Two team members (YAA- a Senior Specialist in Infectious Diseases and JA- an experienced Public Health Specialist) did the initial evaluation of the IACS tool for scabies diagnosis and subsequently provided training on the use of the tool to the other members of the assessment team. All trainings were under the supervision of ROP, T-vdW and YS who are Professors of Infectious Diseases with many years of experience in the field.

We have clarified by inserting the following (line 98-102): ‘The assessment team consisted of medical doctors with clinical experience diagnosing scabies based on earlier activities in infectious diseases and/or public health. In addition, a supplemental training program on the diagnosis of scabies, impetigo and other locally common skin conditions as well as the use of the IACS criteria for scabies diagnosis was provided to team members.’

Samples from two populations were looked for, community members via house-to-house visits (within which local boundaries?) and school children via a random selection. What is meant with “.. all persons present ..” (line 101)? Present where? Some ideas of what constitutes the numerators are needed.

Response: We have revised this line to make the content clearer. The line; ‘Per community, a house to house visit was performed till all persons present were invited to participate’ has been changed as follows (line 104-105): ‘Per community, a house to house visit was performed. All persons present in the house at the time of the visit were invited to participate.’

The REDCap questionnaire needs a better description and at least a reference.

Response: We have updated the methods section to provide further details on the REDCap questionnaire as follows (line 115-124): ‘A focused history of standardized questions was taken of all participants consisting of information required for the IACS Criteria classification. Questions included whether participants experienced itch. Contact history was assessed by asking if participants lived with someone, or had a friend or classmate with itch, or if they lived with someone, or had a friend or classmate with a rash that looks like scabies. Participants were shown images of people with typical scabies rashes to assist these questions. Questions on treatment included whether participants had received any scabies treatment in the preceding two months, what treatment was received if any and a description of how the treatment was used. History was taken in the local language (with the assistance of interpreters where required) or in English.’

The standardised body examination was made as “in a previous study (7)”. The authors could have been a bit more detailed here and for that used as a good example that reference, a recent school study from the Solomon Islands (Osti MH et al. BMC Infect Dis. 2019): “Impetigo was diagnosed on the presence of papules, pustules or ulcerative lesions with associated erythema, crusting or pus. Skin examination consisted of assessment of exposed areas: the feet and legs to the thighs, hands to the upper arms, neck, face and scalp. Students were in school uniform which consisted of above-knee shorts and above-elbow shirts or dresses. Shoes were removed prior to examination.”  Did the examination procedure differ between school-children and adults?

Response: We have revised the section as suggested. It now reads (line 109-124): ‘Briefly, skin examination consisted of assessment of exposed areas: the feet and legs to the thighs, hands to the upper arms, neck, face and scalp. Students were in school uniform which consisted of above-knee shorts and above-elbow shirts or dresses. Shoes were removed prior to examination. Adults were also required to have the designated body regions exposed prior to their skin examination. The examination excluded breasts and genitals, unless requested by participants and then only in a separate, private examination area. A focused history of standardized questions was taken of all participants consisting of information required for the IACS Criteria classification. Questions included whether participants experienced itch. Contact history was assessed by asking if participants lived with someone, or had a friend or classmate with itch, or if they lived with someone, or had a friend or classmate with a rash that looks like scabies. Participants were shown images of people with typical scabies rashes to assist these questions. Questions on treatment included whether participants had received any scabies treatment in the preceding two months, what treatment was received if any and a description of how the treatment was used. History was taken in the local language (with the assistance of interpreters where required) or in English.’

The assessment of the severity of scabies and impetigo merits some few comments than just a reference.

Response: We have modified by the methods section by adding (line 135-138): ‘Scabies was categorized as: mild, 1 to 10 lesions; moderate, 11 to 49 lesions; or severe, 50 or more lesions. Impetigo was classified as: very mild, 1 to 5 lesions; mild, 6 to 10 lesions; moderate, 11 to 49 lesions; or severe, 50 or more lesions.’

Earlier treatment, how long ago was that asked for?

Response: We enquired about any scabies treatment received in the preceding 2 months. We had indicated this in the submitted manuscript with the statement: ‘At the time of the interview, 119 (59.4%) of the 200 participants with scabies had recently received treatment with topical benzyl benzoate (first line treatment in Ghana) in the past two months because of their skin problems.’ (line 185-188)

Statistical methods should be dealt with under its own heading. You have evidently used descriptive statistics with proportions. How do you deal with the uncertainties? With which methods did you make your analyses (SPSS/IBM does not do your thinking....). In the Results section, line 130, you introduce the acronym IQR without an earlier explanation under the Statistical methods. Your numbers in the different categories are small and the p-values have to be used with caution. Why are confidence intervals not used? Line 156: “The participants   ....similar ..... scabies severity compared to the previously untreated participants (p=0.063).” This statement is not correct. You cannot judge similarity out of a p-value.

Response: We have added a section on ‘Statistical analysis’ to provide details of the statistical methods used by inserting:

‘Statistical analysis

We conducted descriptive and inferential statistical analyses to present the data of the outbreak. Categorical variables were expressed as frequencies and proportions; and results for continuous variables were expressed as median and interquartile range (IQR). The severity of scabies and impetigo in the earlier treated and untreated groups were compared using the Mann-Whitney (MWU) test. The Relative Risk (RR) of impetigo in participants with or without scabies was calculated with 95% CI. Statistical analysis was performed using IBM SPSS statistics Version 20 (IBM Company, Armonk, NY, USA).’ (Line 141-148)

We have also defined IQR as suggested (line 144).

The severity of scabies and impetigo in the earlier treated and untreated groups have been compared using the Mann-Whitney (MWU) test. The section now reads (line 191-194): ‘The participants who were treated in the past months showed similar scabies severity compared to the untreated participants (MWU, p = 0.068). Impetigo seemed less common among previously treated scabies participants; however, this was not statistically significant (MWU, p = 0.25).’
Line 120: Comparisons are made between proportions in subgroups. By “Linear-by-linear analysis” is probably meant an analysis for linear trend in categorical data.
The Results section is difficult to follow due to the lack of the information I have pointed out above.

Response: We have modified the methods to include a section on statistical analysis. The severity of scabies and impetigo in the earlier treated and untreated groups have been compared using the Mann-Whitney (MWU) test as indicated in the methods section.

In SPSS statistical tool, the Mantel-Haenszel (MH) test is sometimes referred to as linear-by-linear analysis; we have deleted this as the MH test statistic was no longer utilised.

Does “Missing” in Table 1 mean ‘Missing information’ or ‘dropped out’?

Response: Missing information. This has been revised accordingly

Figure 2. “.... some participants.” = Four individuals, children, adults?

Response: The figure title has been modified to clarify this. The legend now reads:

‘*Scabies lesions seen on exposed body regions in four selected participants’*

Table 2. The * is missing in the table.

Response: We were defining the IACS categories included in the table. We do not find the use of the * necessary and have deleted it

Discussion and conclusion.
Line 170: “Here, we report......”. Already said, could be removed. As well as line 179: “Published data....”.

Response: Done as suggested
Line 171 onwards: When comparisons with other studies outside Ghana are made, it is important to comment on how these studies were made and whether comparisons are really valid.

Response: We have added a section on study limitations to address the concern raised by the reviewer.
Line 92: Are kidney diseases and rheumatic heart disease strikingly common in Ghana, or in the northern parts of Ghana?

Response: At the present time, there is a dearth of data on the situation in the northern part of Ghana. We hope to set up a follow up study to answer these and other questions regarding the impact of scabies on kidney diseases and rheumatic heart disease in northern Ghana.

The reference list contains 26 items and is up-to-date and comprehensive.

Reviewer #2: This is an important study on scabies in Ghana as there is limited data on scabies prevalence in much of West Africa but it requires substantial work to improve the description of the study methodology and the reporting of the results.

1) In the abstract you say that scabies can be associated with RhD/RhF; i think the wording in the introduction where you say 'possibly' is more accurate and the abstract should be amended to be in line with the main text.

Response: The section of the Author summary has been revised. It now reads (line 35-37): ‘Secondary bacterial infections following scabies can lead to significant complications including chronic kidney disease from glomerulonephritis and possibly rheumatic heart disease.’

2) Line 65 - you say that fomites may play a role; this statment is relatively controversial as many would argue that fomites play a minimal role in transmission especially in LMIC/highly endemic settings. At a minimum this statement will need a reference.

Response: Admittedly, most scabies transmission is by direct skin-to-skin contact including sexual contact. Although less common, contact with infested fomites (e.g. clothing, bedding and towels) have been thought to play a role. References have been inserted to support the statement. The section now reads (line 66-68): ‘Scabies transmission is predominantly by direct contact (including sexual contact) with infected skin (5, 6). Although less common, contact with infested fomites including clothing, bedding and towels have been thought to play a role (5, 7).’

3) Im not sure that line 76 adds anything - scabies is found/endemic everywhere, and having referenced 2 studies confirming scabies in Ghana I am not sure what is added by saying that scabies is endemic in Ghana.

Response: We have deleted the statement ‘There is anecdotal evidence that scabies is endemic in Ghana.’

4) Line 79 - please provide more details. Was treatment limited strictly to cases or also their contacts? What treatment was provided by the municipal health authorities.

Response: We indicated that the district health authorities treated individual patients. We have clarified that individual patients received topical benzyl benzoate. The section now reads (line 80-81): ‘The district health team treated individual patients with scabies within the communities with topical benzyl benzoate.’

5) Methods - the sampling frame needs a clearer description. How many communities were visited? What proportion of houses were visited?

Response: Please see response to reviewer 3 above.

6) Line 103/4 I think you mean was recorded in a REDCAP database rather than obtained as presumably information was obtained by history/examination.

Response: Agreed. This has been corrected and the methods section has been revised accordingly (line 106).

7) Critically important is to understand is who conducted the examinations, what training they had received and how you confirmed they could correctly identify lesion morphology and distribution in line with the IACS criteria.  This is not currently described and must be added.

Response: We have updated the methods section to include the following sentences (line 98-102): ‘The assessment team consisted of medical doctors with clinical experience diagnosing scabies based on earlier activities in infectious diseases and/or public health. In addition, a supplemental training program on the diagnosis of scabies, impetigo and other locally common skin conditions as well as the use of the IACS criteria for scabies diagnosis was provided to team members.’

8) I presume children did not provide written informed consent but instead this was obtained from a parent/guardian. Please clarify.

Response: We have clarified this by revising the section. Parents and guardians provided consent for younger children but children in the senior high school gave assent for their inclusion after permission had been obtained from the school authorities. We have revised the ethics section to include the statement (line 151-154): ‘For young children within the communities, consent was obtained from parents or legal guardians. In the school, in addition to obtaining permission from the school authorities, the school children who were < 18 years provided assent for their inclusion in the study.’

9) In the results and in line with the comment about sampling frame it is very unclear what % of individuals in the 4 communities were enrolled. Was it all residents? If not were there any systematic issues in sampling (i note for example that 61.5% of participants were female which suggests systematic bias in to study inclusion).

Response: Please see response to reviewer 3 above.

In addition, we have included a section on study limitations (line 320-333) which reads: ‘This was an opportunistic assessment that was undertaken in the context of investigation of an outbreak situation in Northern Ghana. The sample size of this study is relatively small compared to the population of the entire district. Even though there may be differences in scabies prevalence between communities in the district, due to the close person-to-person interactions between communities (resulting from close extended family ties), it is unlikely that such a percentage would be lower than 10% (MDA indicated if higher) considering the high percentage of scabies found in the communities studied. The students studied come from different communities across the district. Furthermore, the district health team ended up providing MDA across the district and did not observe communities without scabies problems. This study provides useful data on scabies burden and could form the basis for guiding future research in Ghana and West Africa where there is a dearth of data on scabies prevalence. The IACS criteria for standardisation of scabies diagnosis was easily and practically applied in our field survey in a resource limited setting in rural Ghana.’

10) The use of the IACS criteria needs revising. The authors state LINE 137 that a large proportion of patients had burros but then these individuals should be classified as B1 not B3. Please revise the methods and results accordingly.

Response: This is indeed so. We have revised Table 2 accordingly.

11) It would be useful to show some association data for scabies and impetigo as there is a lack of clarity on whether the strong relationship shown in the Pacific is seen elsewhere. This could be for example by including an odds ratio for impetigo amongst those with scabies and/or calculating the population attributable fraction of impetigo due to scabies.

Response: We have inserted the following sentences in the results section (line 197-198): ‘Impetigo was found in 22% (44/200) of participants with scabies and 10.8% of those without scabies [RR 2.27 (95% CI 1.21- 4.27)].’

12) It would be useful also to show scabies (and impetigo) prevalence stratified by key variables such as age and gender because rates often vary markedly by age group; given the non representative sampling methodology this is important to understand.

Response: We have inserted the following statement in the results section (line 199-200): ‘Scabies was commoner in participants ≤16 years compared to those >16 years [RR 3.06 (95% CI 1.73-5.45)].’

13) I would be careful comparing the prevalence found in this study, which deliberately targeted an area where there was high reporting of scabies cases, with representative sampling data. The limitations of these comparisons should be noted.

Response: We have added a section on the limitations of the current study (320-333).

14) New results are presented in the discussion (see for example line 197-198) - please avoid this by including relevant information in the results section.

Response: We deleted the sentence ‘Ninety five of 200 (47.5%) scabies patients were aged ≤ 18 years’.

15) Line 208 ivermectin doesn’t need a capital letter

Response: Corrected as suggested (line 270).

16) Paragraph beginning line 203; this section should clearly discuss the already existing evidence that treatment of index cases alone is associated with much higher rates of reinfestation; this is why (even outside the context of MDA) almost all guidelines recommend treating the whole house/close contacts.

Response: We have inserted the following sentences (line 298-306) in the discussion section to address the reviewer’s comment: ‘A control strategy of treating clinical cases and their contacts undoubtedly provides relief for individuals with scabies, but its success in reducing population prevalence in the longer term is limited (33). Mass drug administration (MDA) using topical permethrin or oral ivermectin offers an alternative approach for population control to substantially reduce the burden of scabies. Among populations of northern Australia (36, 37), Egypt (15) and in Panama (38), mass treatment of highly endemic communities with topical 5% permethrin substantially reduced scabies prevalence. In another study with permethrin, although the scabies prevalence remained unchanged, the prevalence of infected scabies decreased from 3.7% to 1.5% representing a relative reduction of 59% (39).’

Reviewer #3: (No Response)

Again, we thank the Editors for the consideration given to our manuscript and count on your kind assurances.

Yours Sincerely,

Yaw Ampem Amoako

Corresponding Author
