# Supplementary material for: A scabies outbreak in the North East Region of Ghana: The necessity for prompt intervention
Source: PLoS Negl Trop Dis. 2020 Dec 22;14(12):e0008902. doi: 10.1371/journal.pntd.0008902 (PMC7787682; doi:10.1371/journal.pntd.0008902)
Supplement: Attachment — Submitted filename: Response to review comments R2 31 07 2020.docx [file pntd.0008902.s003.docx]

31^st^ July, 2020

The Editor-in-Chief

PLOS Neglected Tropical Diseases

Dear Sir,

**Re: Decision on your PLOS Neglected Tropical Diseases submission (PNTD-D-20-00305R1) - [EMID:71b0bf53fdd02580]"A scabies outbreak in the North East Region of Ghana: the necessity for prompt intervention"**
 
My co-authors and I will like to thank the editors and reviewers for the feedback on our manuscript. We have taken note of the review comments and wish to respond as follows:

Reviewer's Responses to Questions

Key Review Criteria Required for Acceptance
As you describe the new analyses required for acceptance, please consider the following:

Methods
-Are the objectives of the study clearly articulated with a clear testable hypothesis stated?
-Is the study design appropriate to address the stated objectives?
-Is the population clearly described and appropriate for the hypothesis being tested?
-Is the sample size sufficient to ensure adequate power to address the hypothesis being tested?
-Were correct statistical analysis used to support conclusions?
-Are there concerns about ethical or regulatory requirements being met?

Reviewer #1: The paper has been edited and is somewhat easier to follow, but there are still shortcomings of different kinds that need to be taken care of before it is publishable.
Comments and suggestions that may be of help for the revision:
The Title is not covering the contents of the article. Suggested key words for the title are scabies, outbreak, prevalence, mass drug administration.

Response: We have investigated a scabies outbreak in northern Ghana and found a high prevalence of 71%. Our study calls for a mass drug administration in this outbreak setting but does not describe the mass drug administration itself. We therefore chose not to include the key word ‘mass drug administration’ in the title, not to mislead the readers. We hope the editor can advise what is desirable in this case.

The Abstract needs to be rewritten. It is not giving a good and easy-to-read overview of the article.

Response: We have rewritten the abstract as suggested. It now reads as follows:

‘Abstract

Background

There is a dearth of data on scabies from Ghana. In September 2019, local health authorities in the East Mamprusi district of northern Ghana received reports of scabies from many parts of the district. Due to on-going reports of more cases, a medical team visited the communities to assess the effect of the earlier individual treatment on the outbreak. We furthermore aimed to contribute to the data on scabies burden in Ghana and to assess the practicality of using the International Alliance for the Control of Scabies (IACS) diagnostic tool in a field survey in a resource limited setting.

Methodology/ Principal Findings

This was a prospective cross sectional study. Demographic information and medical history was collected on all participants using a REDCap questionnaire. A standardised skin examination of exposed regions of the body was performed on all participants. Scabies was diagnosed based on the criteria of the International Alliance for the Control of Scabies (IACS). Participants were mostly female (61.5%) and had a median age of 18.8 years (IQR 13-25). Two hundred out of 283 (71%) of participants had scabies with most (47%) presenting with moderate disease. Impetigo was found in 22% of participants with scabies and 10.8% of those without scabies [RR 2.27 (95% CI 1.21- 4.27)]. 119 participants who received scabies treatment in the past months still had clinical evidence of the disease. 97% of participants reported a recent scabies contact. Scabies was commoner in participants ≤16 years compared to those >16 years [RR 3.06 (95% CI 1.73-5.45)].

Conclusion/ Significance

The prevalence of scabies was extremely high. The lack of a systematic approach to scabies treatment led to recurrence and ongoing community spread. The IACS criteria was useful in this outbreak assessment in Ghana. Alternative strategies such as Mass drug administration may be required to contain outbreaks early in such settings.’

 If the Introduction could begin like this “Scabies is an intensely pruritic skin disease caused by the mite....//persons (1).”, you would avoid the value-laden word “immense”.

Response: Done as suggested
Line 50: A more correct way of using uppercase letters and the acronym: “making scabies one of the most common neglected tropical diseases (2). Recently, the WHO has classified scabies as a Neglected Tropical Disease (NTD) on the .....”

Response: Done as suggested
Line 55: instead of “acute and secondary complications”,  “acute symptoms and secondary complications” is more logical.

Response: Done. We have inserted the word ‘symptoms’ after acute as suggested. Section now reads: ‘Scabies and its associated acute symptoms and secondary complications pose a significant socioeconomic burden to affected persons, families, communities and the health system.’
Line 73: Better “.... estimated scabies worldwide to range from .....”.

Response: Done as suggested
Line 84: A clear stated aim/objectives is still missing. Since you conclude things at the end you must have had some intentions with your writing up of the data!

Response: We have stated the study aim by inserting the following: ‘We aimed to study the outbreak to contribute to the data on scabies burden in Ghana and to assess the practicality of using the International Alliance for the Control of Scabies (IACS) diagnostic tool in a field survey in a resource limited setting.’
Line 99: The acronym IACS has not been explained before in the bread text and should be made here and not in line 124.

Response: Done
Line 104: The REDcap questionnaire needs a reference!

Response: Done
Line 107: “..... exposed regions of the skin was done in a previous study from Solomon Islands (10). Briefly, the skin examination.......”

Response: Done
Line 115: A table with the IACS criteria would be helpful here.  E.g. with the text from lines174-180, given together with the text from the paragraph in line 123-130.

Response: This has been done as suggested. The numbering of the tables have been adjusted accordingly.

Table 1 Case definitions for scabies using the IACS criteria

| Clinical category |  | Used in survey |
| --- | --- | --- |
| Confirmed scabies  A1  A2  A3 | Mites, eggs or faeces on light microscopy of skin samples  Mites, eggs or faeces visualised on individual using high powered imaging device  Mite visualised on individual using dermoscopy | No  No  No |
| Clinical scabies  *B1  B2  B3 | Presence of burrows  Typical lesions affecting male genitalia  Typical lesions in a typical distribution and two history features (itch and contact history) | Yes  No  Yes |
| Suspected scabies  C1  C2 | Typical lesions in a typical distribution and one history feature (itch or contact history)  Atypical lesions or atypical distribution and two history features (itch and close contact with an individual who has itch or typical scabies lesions in a typical distribution) | Yes  Yes |

**Burrows were not confirmed with dermoscopy*

Line 138: The standard protocol in Ghana needs a reference.

Response: Done
Line 144-145: Don’t introduce an acronym for the Mann-Whitney test. It is not needed. The relative risk (RR) of .....//. .... calculated with 95% confidence interval (CI). Level of significance?

Response: We have deleted the MWU acronym for the Mann-Whitney test. We also inserted the sentence ‘A p value <0.05 was set as the level of statistical significance’ as suggested.
Line 152: “...<18 years provided assent for their...”. A bit unclear. Assent from whom?

Response: In Ghana, consent may only be given by individuals who have reached the legal age of consent (this is typically 18 years). Assent is the agreement of someone not able to give legal consent to participate in the study (in this study, school children under 18 years gave assent).
Line 164: * Other instead of “other”.

Response: Done
Line 190: The statement here is still wrong!! You cannot write “similar scabies severity”! You cannot judge similarity out of a p-value. You do not need MWU here, just the p-value.

Response: Revision done. the sentence now reads: ‘There were no statistically significant differences in the severity of scabies between the participants who were treated in the past months when compared to the untreated participants (p = 0.068).’
Line 193: .... treated for scabies were free...... Remove the comma.

Response: Done
Line 195: remove (44/260). The numbers are in the tables and the percentage will suffice here.

Response: Done
Line 196-197: 119 participants...... The sentence is a bit elliptic. Should also begin with “One-hundred-and-nineteen” to be consequent with what is done in Line 231: Eighty three......

Response: The sentence now reads ‘One hundred and nineteen (119) participants who had been previously treated still had clinical evidence of scabies.’
Line 221-222: Scabies predominantly affects ......  Already said, does not need to be repeated.

Response: We have deleted the sentence ‘Scabies predominantly affects disadvantaged people in low- and middle-income countries’ as suggested.
Line 227-229: Remove sentence since it is already said and you have not studied the occurrence of the medical complications.

Response: Done as suggested.
Line 252: Better “This is similar to the epidemiology of other NTDs of the skin like Buruli ulcer (27,28) and Yaws (29).”.

Response: The section has been revised as per the suggestion of the reviewer. It now reads: ‘Scabies affected predominantly young persons in this study. Most patients with scabies were aged ≤16 years. This is similar to the epidemiology of other NTDs of the skin like Buruli ulcer (27, 28) and Yaws (29).’
Line 257: Better “Treatment options for scabies in Ghana include......”

Response: Done as suggested.
Line 303: Do you mean the prevalence of secondary infected scabies? Unclear sentence.

Response: We have inserted ‘secondary’ between infected and scabies
Line 325: Better “The examined students come from .......”

Response: Done
Line 328-331: Give these sentences a heading of its own, “Conclusion”

Response: Done
Reviewer #2: (No Response)
Reviewer #3: (No Response)

Results
-Does the analysis presented match the analysis plan?
-Are the results clearly and completely presented?
-Are the figures (Tables, Images) of sufficient quality for clarity?

Reviewer #1: Please, see comments under Methods!
Reviewer #2: (No Response)
Reviewer #3: (No Response)

Conclusions
-Are the conclusions supported by the data presented?
-Are the limitations of analysis clearly described?
-Do the authors discuss how these data can be helpful to advance our understanding of the topic under study?
-Is public health relevance addressed?

Reviewer #1: Please, see comments under Methods!
Reviewer #2: (No Response)
Reviewer #3: (No Response)

Editorial and Data Presentation Modifications?
Use this section for editorial suggestions as well as relatively minor modifications of existing data that would enhance clarity. If the only modifications needed are minor and/or editorial, you may wish to recommend “Minor Revision” or “Accept”.

Reviewer #1: Please, see comments under Methods!
Reviewer #2: (No Response)
Reviewer #3: (No Response)

Summary and General Comments
Use  this section to provide overall comments, discuss strengths/weaknesses of the study, novelty, significance, general execution and scholarship. You may also include additional comments for the author, including concerns about dual publication, research ethics, or publication ethics. If requesting major revision, please articulate the new experiments that are needed.

Reviewer #1: Please, see comments under Methods!
Reviewer #2: I am satisfied the comments have been addressed
Reviewer #3: Well done – a much improved manuscript – minor comments below

Abstract
-       Specify study design - cross sectional study to assess prevalence

Response: Done
-       tighten paragraph on methods line 11-18 – could be shortened to 1-2 sentences. (important to mention skin assessment and IACS criteria here but less other details required)

Response: Done. We have revised the abstract.
-       specify timing of treatment (recent treatment for scabies?) / exposure (how many weeks? (or could use “recent”) – may be easier to say “97% of participants reported a recent scabies contact”

Response: Revised as suggested
-       temper the conclusion re MDA – this study doesn’t demonstrate anything about efficacy of MDA – perhaps “Alternative strategies such as MDA may be required to contain outbreaks in such settings” or something regarding measuring efficacy

Response: Done
-       revise sentence on MDA in Author’s summary section (as per comment above)

Response: Done
Methods
-       as per comment in abstract – specify type of study (?prospective cross-sectional)

Response: Done as suggested.
Results
-       quantivy “much less” line 194 ?comparison done

Response: We have revised the section to now read: ‘Even in participants without clinically evident scabies, itch and rash were common (40.9% and 26.5% respectively) but this was much less than in those with scabies (95% and 95.5% respectively) as shown in Table 4.’
Discussion

Reviewer #1: No
Reviewer #2: No
Reviewer #3: No

Again, we thank the Editors for the consideration given to our manuscript and count on your kind assurances.

Yours Sincerely,

Yaw Ampem Amoako

Corresponding Author
