# Supplementary material for: A scabies outbreak in the North East Region of Ghana: The necessity for prompt intervention
Source: PLoS Negl Trop Dis. 2020 Dec 22;14(12):e0008902. doi: 10.1371/journal.pntd.0008902 (PMC7787682; doi:10.1371/journal.pntd.0008902)
Supplement: Attachment — Submitted filename: Response to reviewer R4.docx [file pntd.0008902.s005.docx]

13^th^ October, 2020

The Editor-in-Chief

PLOS Neglected Tropical Diseases

Dear Sir,

**Decision on your PLOS Neglected Tropical Diseases submission (PNTD-D-20-00305R3) - [EMID:2da0cab22642ec8a] ‘’A scabies outbreak in the North East Region of Ghana: the necessity for prompt intervention"**

My co-authors and I will like to thank the editors and reviewers for the feedback on our manuscript. We have taken note of the review comments and wish to respond as follows: 
 
Reviewer's Responses to Questions

Key Review Criteria Required for Acceptance?
As you describe the new analyses required for acceptance, please consider the following:

Methods
-Are the objectives of the study clearly articulated with a clear testable hypothesis stated?
-Is the study design appropriate to address the stated objectives?
-Is the population clearly described and appropriate for the hypothesis being tested?
-Is the sample size sufficient to ensure adequate power to address the hypothesis being tested?
-Were correct statistical analysis used to support conclusions?
-Are there concerns about ethical or regulatory requirements being met?

Reviewer #1: (No Response)

Reviewer #2: (No Response)

Reviewer #3: no concerns

Results
-Does the analysis presented match the analysis plan?
-Are the results clearly and completely presented?
-Are the figures (Tables, Images) of sufficient quality for clarity?

Reviewer #1: (No Response)

Reviewer #2: (No Response)

Reviewer #3: no concerns

Conclusions
-Are the conclusions supported by the data presented?
-Are the limitations of analysis clearly described?
-Do the authors discuss how these data can be helpful to advance our understanding of the topic under study?
-Is public health relevance addressed?

Reviewer #1: (No Response)

Reviewer #2: (No Response)

Reviewer #3: no concerns

Editorial and Data Presentation Modifications?
Use this section for editorial suggestions as well as relatively minor modifications of existing data that would enhance clarity. If the only modifications needed are minor and/or editorial, you may wish to recommend “Minor Revision” or “Accept”.

Reviewer #1: (No Response)

Reviewer #2: (No Response)

Reviewer #3: no concerns

Summary and General Comments
Use  this section to provide overall comments, discuss strengths/weaknesses of the study, novelty, significance, general execution and scholarship. You may also include additional comments for the author, including concerns about dual publication, research ethics, or publication ethics. If requesting major revision, please articulate the new experiments that are needed.

Reviewer #1: The manuscript has benefitted from the revisions, but I still have some comments:
Line 84: “assess the practicality” implies a scientific approach. Your assessment, however, seems to be based on the subjective judgements by the team members.

Response: The section has been revised and now reads: ‘The assessment team aimed to study the outbreak to contribute to the data on scabies burden in Ghana and to demonstrate the use of the International Alliance for the Control of Scabies (IACS) diagnostic tool in a field survey in a resource limited setting.’

Line 106: The REDCap mobile app is not wellknown enough to be presented like that. I strongly recommend that the questionnaire is added as a Supplementary file to the manuscript.

Response: The questionnaire has been added as a supplementary file to the manuscript.

Line 226: “MDA” is mentioned for the first time in the bread text and therefore has to be explained, i.e. “ mass drug administration (MDA)”.

Response: Done

Reviewer #2: I believe the authors have responded reasonably to the comments from the peer reviewers
Response: We thank the reviewer for the useful comments during the peer review process for this manuscript.

Reviewer #3: I am satisfied with this revised version of the manuscript
Response: We thank the reviewer for the useful comments during the peer review process for this manuscript.

Again, we thank the Editors for the consideration given to our manuscript and count on your kind assurances.

Yours Sincerely,

Yaw Ampem Amoako

Corresponding Author
