# Supplementary material for: A scabies outbreak in the North East Region of Ghana: The necessity for prompt intervention
Source: PLoS Negl Trop Dis. 2020 Dec 22;14(12):e0008902. doi: 10.1371/journal.pntd.0008902 (PMC7787682; doi:10.1371/journal.pntd.0008902)
Supplement: Attachment — Submitted filename: reponse to reviewer comments R3.docx [file pntd.0008902.s004.docx]

8^th^ September, 2020

The Editor-in-Chief

PLOS Neglected Tropical Diseases

Dear Sir,

**Decision on your PLOS Neglected Tropical Diseases submission (PNTD-D-20-00305R2) - [EMID:b85523758982ac86] ‘’A scabies outbreak in the North East Region of Ghana: the necessity for prompt intervention"**

My co-authors and I will like to thank the editors and reviewers for the feedback on our manuscript. We have taken note of the review comments and wish to respond as follows:

Reviewer's Responses to Questions
Key Review Criteria Required for Acceptance?
As you describe the new analyses required for acceptance, please consider the following:

Methods
-Are the objectives of the study clearly articulated with a clear testable hypothesis stated?
-Is the study design appropriate to address the stated objectives?
-Is the population clearly described and appropriate for the hypothesis being tested?
-Is the sample size sufficient to ensure adequate power to address the hypothesis being tested?
-Were correct statistical analysis used to support conclusions?
-Are there concerns about ethical or regulatory requirements being met?

Reviewer #1: See below.

Reviewer #2: (No Response)

Reviewer #3: no concerns

Results
-Does the analysis presented match the analysis plan?
-Are the results clearly and completely presented?
-Are the figures (Tables, Images) of sufficient quality for clarity?

Reviewer #1: See below.

Reviewer #2: (No Response)

Reviewer #3: Results
- would be useful to include comparison of diagnostic classification between treated and non-treated groups - it looks like there is a difference between those with B3 and B1 diagnoses respectively (is this statistically significant?)
- if significant, this raises whether those with B3 diagnoses in the treated group may have resolving scabies (rather than re-infection) - worth including in the discussion as below
Response: Done. We have inserted in the results section the sentence ‘Compared to participants previously treated with benzyl benzoate, scabies burrows were more prevalent among untreated participants. In a post-hoc analysis, there was a statistically significant difference in B3 and B1 diagnostic classification between the participants who were treated with benzyl benzoate in the past months and untreated participants (p < 0.05).’

The discussion section has also been modified as indicated below.
Conclusions
-Are the conclusions supported by the data presented?
-Are the limitations of analysis clearly described?
-Do the authors discuss how these data can be helpful to advance our understanding of the topic under study?
-Is public health relevance addressed?

Reviewer #1: See below.

Reviewer #2: (No Response)

Reviewer #3: Discussion
- line 275 - although resolution of symptoms may take up to 4 weeks (<https://www.nejm.org/doi/10.1056/NEJMcp052784>)
 Response: Done. The reference indicated had already been cited as reference 7 in the manuscript.

- line 280 - revise this statement to consider limitations of timing of review post intervention
- perhaps something along the lines of:
"It is possible that some previously treated patients with an IACS classification of B3 may have resolving scabies rather than re-infection; the lower prevalence of burrows in the treated group compared with those not treated would support this possibility. However, given the timing of reassessment >2-3 weeks after, resolution of symptoms in the majority of treated patients would be expected (reference from benzoate trial by chisodow - <https://www.who.int/bulletin/volumes/87/6/08-052308/en/>)
Response: Done as suggested. The section now reads ‘It is possible that some previously treated patients with an IACS classification of B3 may have resolving scabies rather than re-infection; the lower prevalence of burrows in the treated group compared with those not treated would support this possibility. However, given the timing of reassessment from 3 weeks to 2 months after treatment, resolution of symptoms in the majority of treated patients would be expected (38).’
Editorial and Data Presentation Modifications?
Use this section for editorial suggestions as well as relatively minor modifications of existing data that would enhance clarity. If the only modifications needed are minor and/or editorial, you may wish to recommend “Minor Revision” or “Accept”.

Reviewer #1: See below.

Reviewer #2: (No Response)

Reviewer #3: no concerns - minor revisions

Summary and General Comments
Use  this section to provide overall comments, discuss strengths/weaknesses of the study, novelty, significance, general execution and scholarship. You may also include additional comments for the author, including concerns about dual publication, research ethics, or publication ethics. If requesting major revision, please articulate the new experiments that are needed.

Reviewer #1: Unfortunately, there are still a lot to be criticized even after the second revision. My recommendation is that you re-write the paper and then resubmit it. An analysis of the scabies outbreak in Ghana is of public health interest but needs a more stringent presentation.
Some suggestions:
The Abstract needs to be rewritten, preferably after the bread text has been finished.
Lines 7-8: Is “we” = “a medical team” = “an assessment team”.

Response: Done. For uniformity, we have used ‘assessment team’ throughout the manuscript.
According to the text in lines 8-11 your methodology seems to be both quantitative (prevalence data and impact of earlier treatment) and qualitative ( ..”assess the practicality of using IACS....”).

Response: This was a cross sectional study making use of the IACS diagnostic criteria. No specific qualitative data were collected. The IACS diagnostic criteria enabled the assessment team to systematically examine many people in the community. We therefore changed the text in the methods (lines 9-12) as follows:

‘The assessment team furthermore aimed to contribute to the data on scabies burden in Ghana and to demonstrate the use of the International Alliance for the Control of Scabies (IACS) diagnostic tool in a field survey in a resource limited setting’.

Line 13: “...a prospective cross sectional study..”.  The designation is not according to correct use of epidemiological concepts.

Response: We thank the reviewer for noticing. The word ‘prospective’ has been deleted
Line 50-51: “....making scabies one of the most common neglected tropical diseases”. Remove “neglected”.

Response: We understand the reviewers point. But neglected tropical diseases are 21 and we can based on the literature safely state that scabies is one of the most common of these 21 diseases. It is more complicated to state scabies is one of the most common tropical diseases since we would not know exactly which diseases to count as tropical diseases and which not. We therefore decided to keep the word Neglected.

Line 52: For syntactic reasons remove “on the basis of emerging evidence //... mass drug administration (MDA) and”.

Response: Done
Line 63: S. pyogenes instead of Streptococcus pyogenes, which is already introduced in line 61.

Response: Done
Line 71: remove “described as a disease of poverty and overcrowding and has been”.

Response: Done. Sentence now reads ‘Scabies has been reported to be associated with household size, low socioeconomic status and poor access to healthcare’.
Line 74-76. The sentence is too elliptic.

Response: We deleted the sentence ‘Published data on the prevalence of scabies in Ghana is limited.’

Line 84-89. Be more clear and precise about the aims and objectives. You are presenting prevalence data from a scabies outbreak, essentially a descriptive approach. Then you are comparing groups by age, sex, occupation, earlier treatment and existence of impetigo. This is the quantitative approach. But you are also judging whether IACS was a feasible tool, a qualitative approach.
Response: We have modified the section. No qualitative data was obtained eg by asking the assessment team about their experiences with the IACS criteria. It now reads ‘In this study we report the use of the IACS diagnostic tool which allowed for systematic evaluation of scabies in a field survey in rural Ghana.’

Line 91: please see comment on line 13.

Response: The word ‘prospective’ has been deleted. The section now reads ‘This was a cross sectional study design conducted in the East Mamprusi district which is located in the recently created North East region of Ghana.’
Line 95: The acronym SHS is not needed.

Response: The acronym has been deleted
Line 98. The assessment team needs to be better presented. Is it the same as “we”, “the medical team” etc.?

Response: For uniformity, we have used ‘assessment team’ throughout the manuscript.
Line 101. The acronym IACS is already introduced in line 85.

Response: Done. We have only used the acronym
Line 103: What was randomly selected? Schools, classes, students?

Response: We have already indicated that only one school was involved. We state on line 93-94 that ‘Community members and senior high school students were invited to participate in an interview during house to house visits and a school visit’. As there is only a single senior high school in the district, it was not possible to do a random selection of high schools. We have explained how the selection of student participants in the classes was done. The section reads

‘In the school, there was a random selection of student participants; per classroom, one tenth of the students were invited to participate based on students’ seating in the classroom.’

Line 104: How many communities should be stated here and not just later in the text.

Response: Done. We have added the sentence ‘Five different communities were visited’.

Line 107: The The REDCap reference leads –undated- to a Dutch website, but not to the questionnaire that has been used. A web-based program, that structures your data, is OK, but it does not tell us about how you constructed your questionnaire. The reader must be able to follow an ‘audit trail’ to make up her/his mind of the trustworthiness of the study.
It is of interest to know how many independent variables were used for the analysis.

Response: The reviewer is correct; reference 19 is not helpful. We decided not to add a reference with information on REDCap in general since it is such a well-known program. We described the components of the questionnaire in the methods. If the editor prefers to add the questionnaire; is an appendix with the questionnaire an option?

Results: Use integers for the percentages, but consequently, throughout the entire bread text. Likewise numbers <10 are usually rendered by letters.

Response: Done as requested
Line 165: “2018 IACS criteria”. Why 2018 here and not in the beginning?

Response: Corrected. ‘2018’ has been deleted
Line 169: “crusted scabies” has not been mentioned before.

Response: The sentence ‘The assessment team also looked for crusted scabies’ has been inserted in the methods section.

Line 183: Better to write ‘There was no statistically significant difference in the severity of scabies between the participants, who were treated by ? in the past months and the untreated participants (p=0.068).

Response: Done
Line 185: Remove “Impetigo seemed less common .....”. According to your testing, it was not less common! Why are you doing the statistical testing?

Response: The sentence has been deleted.
Discussion: Must be shortened and more focussed. There are too many repeats. The first sentence could be left out.

Discuss your findings and compare them to other prevalence studies, but don’t write too much about other treatments. It goes too far and beyond the scope of your study.
Line 220 -222 could be left out.

Response: The first sentence ‘A scabies prevalence of 71% was found in the population studied in the East Mamprusi district in the North East region of Ghana’ has been deleted.

The sentences relating to other treatment options for scabies were added in response to previous request made by another reviewer’s comment during a previous round of review.

We have also adjusted text in discussion as follows: ‘The current study has further demonstrated the use of the IACS diagnostic criteria to systematically evaluate for scabies in an outbreak setting in a rural area in Sub-Saharan Africa.’

Due to the changes, the discussion now has the following lines:

‘Patients treated in the preceding 2 weeks might still have symptoms thus making it difficult to distinguish re-infections from primary treatment failure (5, 7, 35). It is possible that some previously treated patients with an IACS classification of B3 may have resolving scabies rather than re-infection; the lower prevalence of burrows in the treated group compared with those not treated would support this possibility. However, given the timing of reassessment 3 weeks to 2 months after treatment, resolution of symptoms in the majority of treated patients would be expected (37).’

Line 223. CI for RR of impetigo...? . “...... implying .//..without scabies” could be left out.
Response: The section has been revised as requested by deleting ‘implying that participants with scabies were twice more likely to have impetigo than those without scabies’. We have inserted the 95% CI for the RR of impetigo as requested

Line 238: ..: “this will be important..... That sentence could be left out.

Response: As requested, we have deleted ‘this will be important to reduce the high burden of scabies infection and subsequent sequelae among populations where scabies infection is endemic’.
Line 280-281: The sentence is a bit cryptic.

Response: The sentence has been deleted.
Line 290. The acronym MDA is already presented in line 53.

Response: Revised. Acronym only used
Line 298:  The word “tremendously” is too emotional to use in a scientific text.

Response: The word ‘tremendously’ has been deleted
Line 311: This sentence belongs to the Introduction and the Method sections.

Response: Done as suggested. The sentence has been moved to the introduction section.
Line 322: Remove “useful” and let the reader decide whether they are useful or not. The data could form a basis for future research.

Response: Done
References:
If a website is referred to, date for your reading it is needed. E.g. Ref 22.

Response: The date of accession for that reference has been added

Reviewer #2: I am satisfied the authors have made the requested amendments.

Reviewer #3: Thank you for the reviewed manuscript - just 2 minor comments regarding assessment in the treated population as outlined above

PLOS authors have the option to publish the peer review history of their article (<a href="<https://journals.plos.org/plosntds/s/editorial-and-peer-review-process#loc-peer-review-history>" target="_blank">what does this mean?</a>). If published, this will include your full peer review and any attached files.

If you choose “no”, your identity will remain anonymous but your review may still be made public.

Do you want your identity to be public for this peer review? For information about this choice, including consent withdrawal, please see our <a href="<https://www.plos.org/privacy-policy>" target="_blank">Privacy Policy</a>.

Reviewer #1: No

Reviewer #2: No

Reviewer #3: No

Again, we thank the Editors for the consideration given to our manuscript and count on your kind assurances.

Yours Sincerely,

Yaw Ampem Amoako

Corresponding Author
